# Use of tricortical iliac crest allograft for augmentation of depressed lateral tibial plateau fracture: a surgical technique

**DOI:** 10.1093/jscr/rjae637

**Published:** 2024-10-17

**Authors:** Pierre-Emmanuel Schwab, Daniel Bravin, Joshua Milby

**Affiliations:** Department of Orthopaedic Surgery, Missouri Orthopaedic Institute, Missouri University Health, 1100 Virginia Avenue, Columbia, MO 65201, United States; Department of Orthopaedic Trauma Surgery, Cox Medical Center South, Missouri University Health, 3801 S National Avenue, Springfield, MO 65807, United States; Department of Orthopaedic Trauma Surgery, Cox Medical Center South, Missouri University Health, 3801 S National Avenue, Springfield, MO 65807, United States

**Keywords:** tibial plateau fractures, allograft, reconstruction

## Abstract

Lateral tibial plateau fractures with significant articular depression and metaphyseal comminution in the setting of osteoporosis are challenging to manage. The subchondral bone defect and capacious cancellous void after surgical elevation of the articular surface is usually filled with nonstructural graft such as autologous cancellous bone graft, allogenic cancellous bone graft, or bone graft substitutes. Reports have shown a high rate of subsidence with these grafts when patients start to bear weight and ultimately failure of the construct. Structural grafts demonstrated stronger mechanical properties and lower subsidence rates. The purpose of this note is to describe a novel surgical technique using structural tricortical iliac crest allograft for the treatment of osteoporotic depressed lateral tibia plateau fracture.

## Introduction

The tibial plateau is the predominantly cancellous region of bone that articulates with the distal femur. It includes the relatively larger, stronger, and more concave medial plateau and the relatively weaker, smaller, and more convex lateral plateau. Lateral plateau injuries may occur from high-energy trauma or from low-energy falls in elderly osteoporotic patients [1]. The direction and magnitude of fracture force in addition to the underlying quality of bone, determine the fracture pattern. Depressed lateral tibial plateau fractures (DLTPFs) can be found in type II-VI fractures according to the Schatzker classification system [2]. To adequately assess the injury, anteroposterior and lateral films should be obtained. Computer tomography scan is helpful in evaluating articular depression and comminution for preoperative planning. Surgical management of DLTPFs involves accurate reconstruction of the articular surface to restore the lower extremity axis with a stable fixation to prevent the development of knee osteoarthritis [3, 4]. After elevation of the articular surface, filling of metaphyseal defect and supplying subchondral support often requires material such as autologous cancellous bone graft, allogenic cancellous bone graft, or bone graft substitutes such as calcium phosphate [5]. Structural grafts have been shown to provide greater mechanical strength compared with the nonstructural grafts [6, 7]. To the best of our knowledge, this if the first time that the use of structural tricortical iliac crest allograft has been reported to fill the metaphyseal void caused by a depression injury of the lateral tibial plateau.

## Surgical technique

### Patient preparation

The patient is identified in the preoperative holding area, and the correct extremity is marked. They are taken to the operating room and a surgical time-out is performed identifying the correct patient, operative site, and procedure to be performed. They undergo general endotracheal anesthesia and are given Cefazolin based on patient’s weight (1 g for patient weighing <80 kg and 2 g for patient weighing >80 kg) and redosed every 4 hours. The patient is placed in a supine position on a radiolucent operating table with the feet brought to the end of the table. Contralateral knee films are obtained with anteroposterior (AP) and lateral views for comparison. A bump consisting of a single rolled blanket is placed behind the operative hip so that the patella is facing forward and helps avoid the leg’s tendency to externally rotate. The injured leg is elevated on a ramp ensuring that visualization of the proximal tibia under fluoroscopy in both the AP and lateral views. A nonsterile tourniquet is applied to the proximal thigh. Typically, a low pressure (250 mmHg) is used to minimize tissue ischemia. Its inflation can be helpful to allow for visualization, particularly of the knee joint. However, it should be limited as much as possible to ensure adequate antibiotic delivery and avoid tissue ischemia. Following the articular reduction, use of the tourniquet can be discontinued. The image intensifier is on the side opposite the injured limb. The entire lower extremity is pre-cleansed with chlorhexidine and alcohol before being prepped from the toes to above the hip using Chloraprep. The lower extremity is draped in a traditional sterile fashion.

### Approach

An anterolateral approach to the proximal tibia centered over Gerdy’s tubercle is used. This path begins 1 cm lateral to the tibial crest distally extending proximally, directly over Gerdys tubercle, to the level of the lateral epicondyle of the femur. The incision is made with a 10-blade, and dissection is carried down using Metzenbaum scissors and Bovie electrocautery. Anterior compartment fascia is incised with a 15-blade 1 cm from the tibial crest extending proximally directly over the Gerdy’s tubercle into the iliotibial band in line with its fibers. Full-thickness flaps are elevated anteriorly and posteriorly off Gerdy’s tubercle providing better exposure and later fascia closure over the plate.

### Reduction

**Figure 1:**
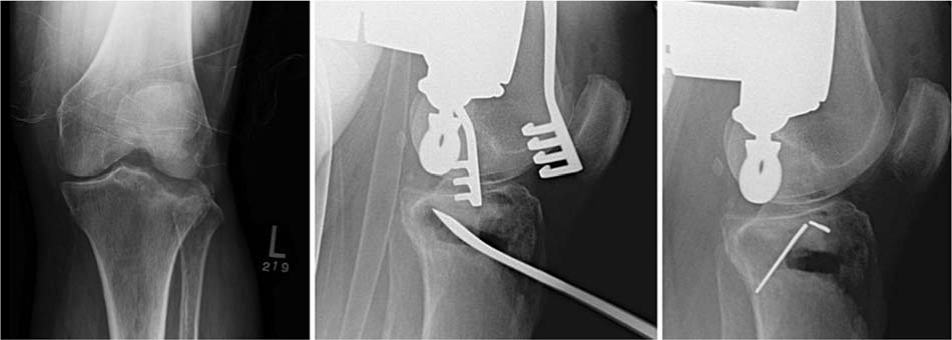
Fracture reduction, articular reduction, posterior slope restoration, and temporary fixation with K-wire.

A 5 × 170 mm Schanz pin is placed percutaneously in the distal tibial shaft after bicortically predrilling with a 3.5 mm drill bit with irrigation. Another 5 × 170 mm Schanz pin is placed parallel to the joint at the level of the lateral epicondyle after bicortically predrilling with a 3.5 mm drill. A universal distractor is placed to enhance joint distraction. A submeniscal arthrotomy is performed parallel to the lateral meniscus to visualize the joint and to inspect the lateral meniscus for tears. Cartilage injury and subchondral bone depression are evaluated as well. The lateral plateau is disimpacted and elevated up using an osteotome deep to the depressed articular surface. Several K-wires are placed lateral to medial through the medial cortex to temporarily hold the alignment of the joint line when it is elevated. The K-wires are pulled out medially until they are flush with the cortex of the lateral plateau. Cancellous allograft is placed under the subchondral bone to reinforce the usually thin articular surface (Fig. 1).

### Tricortical iliac crest allograft preparation and insertion

**Figure 2:**
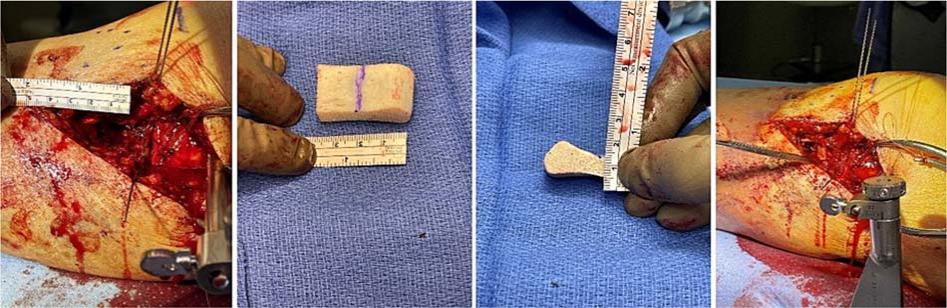
Tricortical iliac crest allograft preparation, measurement, cut to an appropriate size, and insertion into the defect.

The void left in the lateral tibial condyle after elevation of the articular surface is usually triangular in shape. The depth and lateral height are measured. A tricortical iliac crest allograft is selected and cut to an appropriate size based on these measurements. The allograft is then inserted parallel to the joint surface to raft the articular surface. They usually fit nicely thanks to the natural triangular shape of the allograft (Fig. 2).

### Fixation

**Figure 3:**
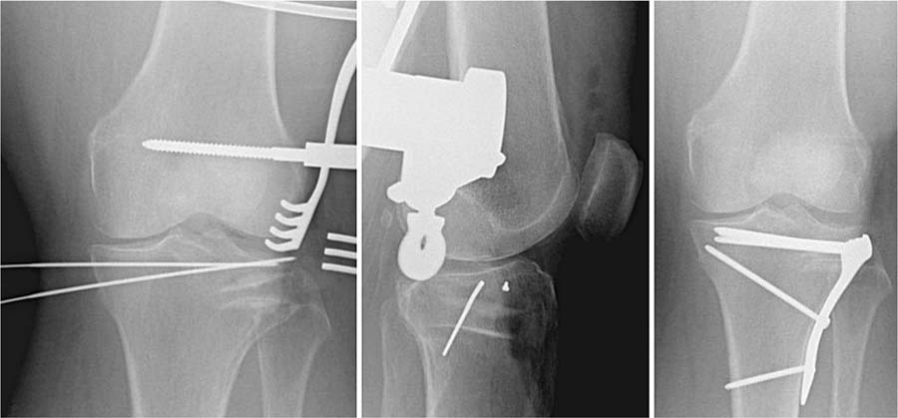
Fracture fixation using 3.5 mm LCP proximal tibia plate, rafting screws, and tricortical iliac crest allograft holding the articular surface.

A 3.5 locking compression plate (LCP) proximal tibia plate is selected and centered on the bone to buttress the lateral condyle and the allograft. The plate is temporarily fixed with two K-wires proximally and distally. When satisfied with the plate position, a 2.5 mm drill bit and a universal drill guide are used to drill bicortically into the axilla, just distal to the apex of the fracture. After measurement of the required length with a depth gauge, a 3.5 mm screw is placed. The same process is repeated in a percutaneous fashion to place a distal 3.5 mm screw through a stab incision. Proximal 3.5 mm locking or non-locking screws, based on surgeon preference and bone quality, are filled using the 2.8 or 2.5 mm drill bit, respectively. The K-wires are removed, and final x-rays are obtained. The medial proximal tibial angle and posterior slope are measured and compared to the contralateral extremity (Fig. 3).

### Closure

Copious irrigation of the surgical wounds with normal saline using cysto tubing is performed. The submeniscal arthrotomy is closed with 0 Vicryl in interrupted figure-of-eight fashion. The IT band and anterior compartment fascia is closed with 0 Vicryl in interrupted figure-of-eight fashion. The subcutaneous layer is closed with 2–0 Vicryl interrupted inverted sutures. The skin is closed with 3–0 nylon in a horizontal mattress fashion. Xeroform, 4×4 gauzes, Webril, and an Ace wrap are used to dress the patient’s wound (Table 1).

**Table 1:** Pearls and pitfalls.

Pearls	Pitfalls
Proper patient position to allow for approach, reduction, and distractor placement in addition to ability to take x-rays with image intensifier Choose appropriate graft size based on defect Take care to not over-reduce the fracture and check with fluoroscopy and compare to anteroposterior and lateral view of the contralateral knee	Over-reduction can lead to varus alignment Under-reduction can lead to valgus alignment Improper positioning can make fracture fixation difficult

### Postoperative care

The patient is non-weightbearing on their lower extremity for ~8 weeks. Leg elevation placed above heart level is recommended postoperatively to minimize swelling and protect the closure of the incision. Anticoagulation is chosen based on medical comorbidities and surgeon preference. Patients are seen for clinic visits at 2 weeks, 6 weeks, 12 weeks, 6 months, and 1 year postoperatively with x-rays at each visit. At the 2–3 week visit the sutures are removed if healing allows. Knee range of motion exercises are initiated. At 6 weeks therapy is initiated for knee range of motion if they are not able to flex past 90 degrees and instructions provided to initiate progressive weightbearing at 8 weeks starting at 25% of their body weight and advancing 25% per week until they reach full weight bearing.

## Discussion

Currently, the gold standard to fill a metaphyseal bone defect caused by DLTPFs and to restore the articular surface is the use of nonstructural grafts with either autologous cancellous bone, allogenic cancellous bone, synthetic bone material, or a mix of these grafts. Nonstructural grafts have however shown to bear a high rate of subsidence comprise between 7.8% and 61.5% depending on the type of graft used [6]. Structural grafts offer the advantage to be more robust than their counterpart and demonstrate a lower rate of subsidence compared with cancellous autograft or calcium phosphate [6–8]. Some authors demonstrated the successful use of fibular shaft allograft in depressed tibial plateau fractures with minimal subsidence of the construct [6, 7, 9]. This technical note describes the use of tricortical iliac crest allograft to fill the void left by a depressed tibial plateau fracture (Table 2). The potential advantages of using this type of graft identified by the authors are a better mechanical strength with minimal subsidence of the articular surface; an augmentation of poor bone stock; an adequate mechanical stability to allow for early knee range of motion; a better fit and fill of the shape of the defect left by the elevation of the joint surface; the availability of the graft; the avoidance of potential complications related to autograft harvesting such as infection, hematoma, nerve injury [10]. The use of structural tricortical iliac crest allograft appears to be a promising option to add to the surgeon armamentarium. This paper paves the way for further investigation into the efficacy of this new technique to treat depressed lateral tibial plateau fractures.

**Table 2:** Advantages and disadvantages of the technique.

Advantages	Disadvantages
More mechanical strength Minimal articular subsidence Allow for early knee range of motion Better fit and fill of the shape of the defect left by the elevation of the joint surface Availability of the graft Avoid autograft complications related to autograft harvesting such as infection, hematoma, nerve injury.	Inadequate cut of the graft may result in a mismatch with the bone defect, risk of malalignment and the need to recut a new graft
